# Knockdown of RAD51AP1 suppressed cell proliferation and invasion in esophageal squamous cell carcinoma

**DOI:** 10.1007/s12672-022-00566-2

**Published:** 2022-10-05

**Authors:** Yang-Yang Hu, Chen-Chao Ma, Kai-Xing Ai

**Affiliations:** Department of Thoracic Surgery, Shanghai Pulmonary Hospital, School of Medicine, Tongji University, No.507, Zhengmin Road, Shanghai, 200433 China

**Keywords:** Esophageal squamous cell carcinoma, RAD51AP1, Prognosis, Proliferation, Invasion

## Abstract

**Background:**

Esophageal cancer is a common malignant tumor of digestive tract with esophageal squamous cell carcinoma (ESCC) being the main histological subtype. This study aimed to identify potential hub gene associated with the pathophysiology of ESCC through bioinformatics analysis and experiment validation.

**Methods:**

Three microarray datasets were obtained from the Gene Expression Omnibus (GEO) database. The overlapping differentially expressed genes (DEGs) were analyzed by GEO2R tool. Gene Ontology (GO) and Kyoto Encyclopedia of Genes (KEGG) pathway analyses were performed to predict the potential functions of DEGs. Nine hub genes were identified using protein–protein interaction (PPI) network and Cytoscape software. We selected RAD51-associated protein 1 (RAD51AP1) for further research because of its poor prognosis and it has not been sufficiently studied in ESCC. The effects of RAD51AP1 on proliferation, apoptosis, migration and invasion of ESCC cells were determined by in vitro functional assays.

**Results:**

RAD51AP1 expression was significantly upregulated in ESCC tissues compared with normal tissues by using The Cancer Genome Atlas (TCGA) database. High expression of RAD51AP1 was associated with worse survival in ESCC patients. RAD51AP1 expression was positively associated with the enrichment of Th2 cells and T helper cells. Furthermore, CCK-8 and colony formation assays showed knockdown of RAD51AP1 inhibited the proliferation of ESCC cells. Flow cytometry analysis indicated knockdown of RAD51AP1 induced cell cycle arrest and apoptosis in ESCC cells. Transwell assay revealed knockdown of RAD51AP1 suppressed the migration and invasion of ESCC cells.

**Conclusions:**

Finally, our results demonstrated that RAD51AP1 silencing significantly inhibited cell proliferation and invasion in ESCC, thereby highlighting its potential as a novel target for ESCC treatment.

## Background

Esophageal cancer represents one of the most common malignancies of upper digestive tract, ranking as the 6th leading cause of cancer-related mortalities all over the world [1]. One of the main histological subtypes is esophageal squamous cell carcinoma (ESCC), which accounts for over 90% of the Chinese esophageal cancer patients [2]. Despite continuous improvements in therapeutic method, the survival time of patients with esophageal cancer remains unsatisfied because of late diagnosis and rapid metastasis [3]. It has been reported that ESCC patients has an overall 5-year survival rate of under 30% [4]. A better exploitation of molecular mechanisms underlying the initiation and development of ESCC may help identify novel therapeutic targets and effective diagnostic biomarkers.

RAD51-associated protein 1 (RAD51AP1) is one kind of accessory protein for RAD51 that can vastly improve the activity of recombinase and stimulate the joint molecule formation, playing a crucial role in the repair of chromosome damage mediated by homologous recombination [5]. The silencing of RAD51AP1 leads to genomic instability and defective homologous recombination in human somatic cells [6]. A number of studies have demonstrated that RAD51AP1 is overexpressed and serves as an carcinogenic role in several tumors, including ovarian cancer [7], lung cancer [8], breast cancer [9], as well as hepatocellular carcinoma [10]. However, the functional role of RAD51AP1 in ESCC progression has not been studied.

Recently, sequencing technology and bioinformatic analyses were increasingly applied in screening genetic alterations, and further discovered potential tumor biomarkers [11]. In this research, 3 microarray datasets were firstly downloaded from the Gene Expression Omnibus (GEO) database, and RAD51AP1 was identified as a hub gene in ESCC. Multiple bioinformatics methods were then used to explore its related functions and molecular mechanisms underlying carcinogenesis. Furthermore, several experimental assays were performed to study the influences of RAD51AP1 on cell growth and metastasis of ESCC. This study initially indicated that RAD51AP1 could act as a novel biomarker for diagnosis and prognosis, and a potential therapeutic target for ESCC.

## Methods

### Bioinformatic analysis

Microarray datasets (GSE20347, GSE29001 and GSE38129) were downloaded from GEO database (https://www.ncbi.nlm.nih.gov/geo/), including 17 ESCC tissues and 17 normal esophageal tissues, 21 ESCC tissues and 24 normal esophageal tissues, 30 ESCC tissues and 30 normal esophageal tissues, respectively.

The GEO2R online tool was used to screen differentially expressed genes (DEGs) between ESCC tissues and normal esophageal tissues with the cutoff criteria of adjusted P-value < 0.05 and |log FC|> 2 [12]. We then calculated and drew custom Venn diagrams (http://bioinformatics.psb.ugent.be/webtools/Venn/) to find common DEGs among 3 datasets. DEGs with logFC > 0 was identified as an up-regulated gene, and DEGs with logFC < 0 was identified as a down-regulated gene. Gene Ontology (GO) functional analysis and Kyoto Encyclopedia of Genes (KEGG) pathway analysis were performed to predict the potential functions of DEGs by using online bioinformatic tool DAVID (https://david.ncifcrf.gov/) [13]. The protein–protein interaction (PPI) network of the identified DEGs was constructed by the STRING database (https://cn.string-db.org/), and was then visualized by the Cytoscape software (version 3.9.0) [14]. Furthermore, the MCODE plugin in Cytoscape was applied to look for modules of PPI network (degree cutoff = 2, node score cutoff = 0.2, max. Depth = 100, and k-core = 2).

The mRNA expression level of RAD51AP1 was validated by using data from The Cancer Genome Atlas (TCGA) database (https:// portal.gdc.cancer.gov/). The association between RAD51AP1 expression and overall survival was assessed by Kaplan–Meier potter (http://kmplot.com). The single-sample gene set enrichment analysis (ssGSEA) was performed to access infiltration levels of 24 immune cells by using GSVA package in R software (version 4.0.3) [15]. Correlations between RAD51AP1 expression and infiltration levels of immune cells were calculated by Spearman correlation analysis.

### Cell culture and transfection

Human ESCC cell lines (KYSE70, KYSE150, TE-1, and EC-1) and esophageal epithelial cell line (Het-1A) were purchased from the American Type Culture Collection. Cell lines were cultured in RPMI 1640 medium (containing 10% FBS, 100 U/mL penicillin, and 100 U/mL streptomycin) in an incubator at 37 °C with 5% CO_2_.

Lentiviral vector-mediated short hairpin RNA (shRNA) targeting RAD51AP1 (5′-AGTGATGGTGATAGTGCTA-3′) and negative control shRNA (5′-TTCTCCGAACGTGTCACGT-3′) were obtained from the GeneChem Corporation (Shanghai, China). The vectors were transfected into KYSE150 and TE-1 cell lines by Lipofectamine 2000.

### RNA extraction and quantitative real-time PCR (qPCR)

Total RNA was extracted by the Trizol reagent (Invitrogen, Shanghai, China) following manufacturer's protocols. RNA concentration was detected by using a spectrophotometer at the wavelength of 260 nm. RNA purity was determined by calculating the ratio of A260/A280, and a purified RNA between 1.8 and 2.0 was used in this study. The RNA was reverse transcribed into cDNA by the M-MLV Reverse Transcription Kit (Promega, USA). The qPCR was conducted by the SYBR Master Mixture kit (Takara, Japan) on LightCycler 480 II real-time PCR instrument (Roche, NJ, USA). The qPCR procedures were as follows: an initial denaturation cycle of 30 s at 95 °C, followed by 40 cycles of 5 s at 95 °C and 30 s at 60 °C, and a final cycle of 15 s at 95 °C, 30 s at 60 °C and 15 s at 95 °C for melting curve analysis. Sequences of primers are as follows: RAD51AP1 forward, 5′-TGGTGGTGTTCAAGGGAAAAG-3′, reverse, 5′-AGGTGCAAAGTCTGGTTCAGT-3′; ACTB forward, 5′- GCGTGACATTAAGGAGAAGC-3′, reverse, 5′- CCACGTCACACTTCATGATGG-3′. Relative expression level was normalized to ACTB, and finally calculated using the 2^−ΔΔCT^ comparative method.

### Western blot assay

Proteins were firstly extracted with the RIPA lysis buffer. Protein concentration was measured using BCA protein assay kit (Pierce, USA). 40 μg of protein sample was separated using 10% SDS-PAGE, followed by being transferred onto the PVDF membrane. After being blocked with 5% skimmed milk at room temperature for 1 h, membrane was next incubated by the primary antibody (anti-RAD51AP1, 1:2000, Abcam, ab88370; anti-β-Actin, 1:1000, Abcam, ab8226) at 4 °C overnight. In addition, membrane was incubated by the horseradish peroxidase (HRP)-conjugated IgG secondary antibody (1:2000, Abcam, ab6721) for 1 h. Results were finally detected by the chemiluminescence detection system.

### Cell proliferation assay

Cell counting kit-8 (CCK-8) assay was conducted to evaluate cell proliferation [16]. Transfected cells were seeded into a 96-well plate at the density of 2 × 10^3^ cells, and cultured for 1, 2, 3, 4, or 5 days. 10 μl CCK-8 reagent was added into every well. The absorbance was finally detected at 450 nm with the microplate reader.

### Cell cycle and apoptosis assays

For the analysis of cell cycle distribution, transfected cells were fixed in ice-cold 70% ethanol overnight, and stained with propidium iodide (PI) added with RNase A for half an hour. Cell cycle was then examined with FACSCalibur flow cytometer (BD Bioscience, CA, USA). Cell apoptosis was detected by using Annexin V Apoptosis Detection kit (eBioscience, CA, USA). Transfected cells were collected, and resuspended in binding buffer. Annexin V-APC was next added into the suspension, and incubated for 15 min. Cell apoptosis was analyzed with the flow cytometry.

### Colony formation assay

Transfected cells were added into the 6-well plate with a density of 1 × 10^3^ cells, culturing in RPMI 1640 medium supplemented with 10% FBS. After observing the colonies (10 days), we terminated the culture. Then the cells were fixed in 4% paraformaldehyde and stained with 0.5% crystal violet. Number of colonies (> 50 cells/colony) were finally counted with the fluorescence microscope (Olympus, Tokyo, Japan).

### Transwell migration and invasion assays

Cell migratory and invasive abilities were detected with the 24-well transwell chamber with 8-μm pore size polycarbonate membrane (Corning, USA) [17]. For cell migration, 1 × 10^5^ cells suspended in 100 µL serum-free RPMI 1640 medium was seeded into upper chamber, while 600 µL medium containing 20% FBS was added to lower chamber. For cell invasion, cells in serum-free medium were seeded into upper chamber coated with Matrigel (BD Bioscience, USA). After 24 h, cells on lower surface of filter were fixed with 4% paraformaldehyde and stained with 0.5% crystal violet. Results were finally counted in 5 randomly fields by using the fluorescence microscope.

### Statistical analysis

Statistical analyses were conducted with SPSS (version 22.0). Data was presented as mean ± SD from 3 independent experiments. The data downloaded from TCGA database was converted to transcripts per million reads (TPM) format. Student’s t-test and one-way ANOVA were performed to compare difference between groups. A P value of less than 0.05 was considered statistically significant.

## Results

### Identification of DEGs

**Fig. 1:**
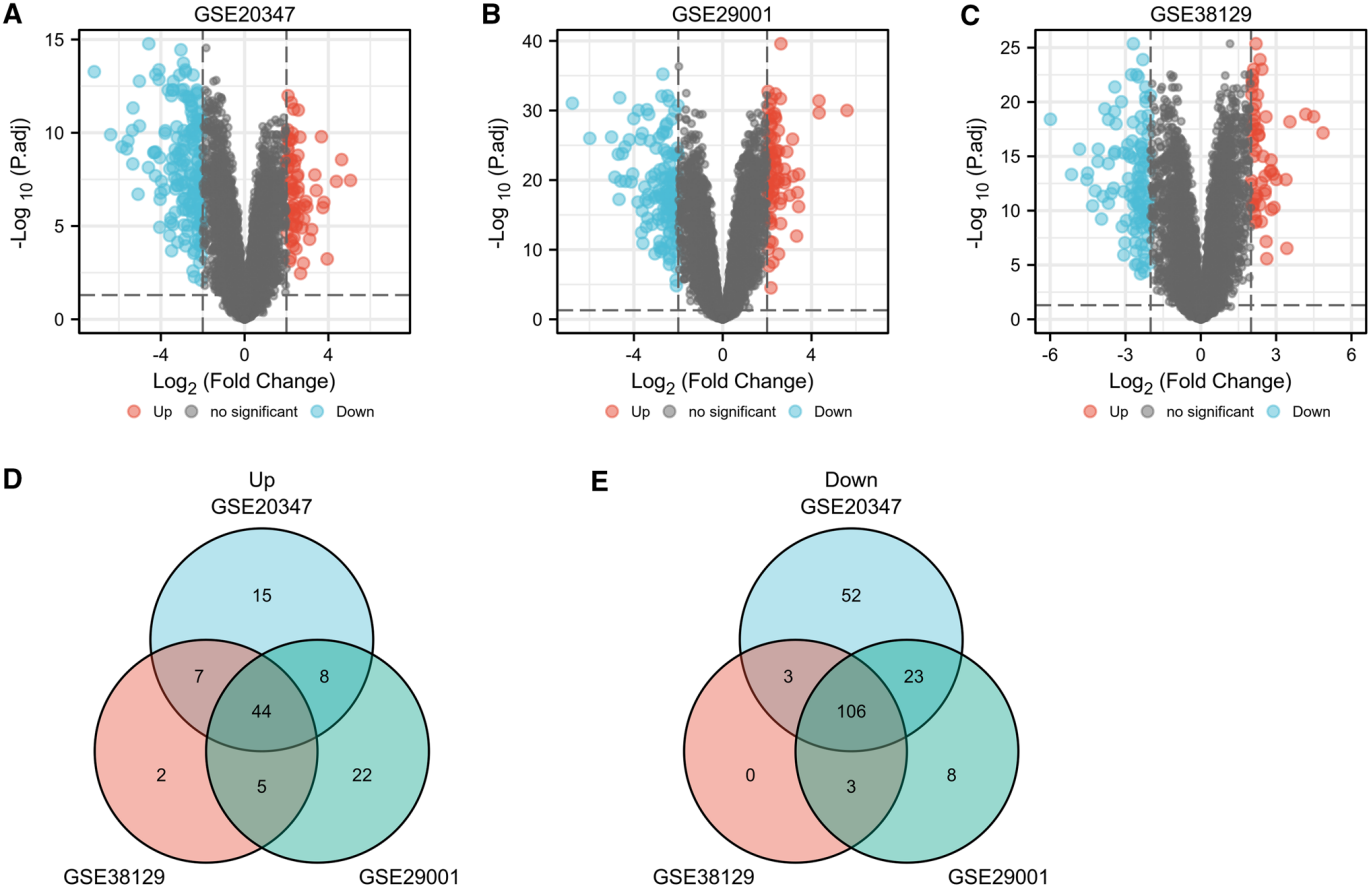
Identification of DEGs. **A**–**C** Volcano plots of DEGs in GSE20347, GSE29001 and GSE38129 datasets. **D**, **E** Venn plots of overlapping DEGs among GSE20347, GSE29001 and GSE38129 datasets. DEGs, differentially expressed genes

We firstly identified DEGs in ESCC through comparing tumor tissues and normal adjacent tissues from GEO database. Volcano plots of DEGs in GSE20347, GSE29001 and GSE38129 were depicted in Fig. 1A–C. Finally, 150 DEGs were found, containing 44 upregulated and 106 downregulated genes (Fig. 1D, E).

### Gene enrichment analyses of DEGs

**Fig. 2:**
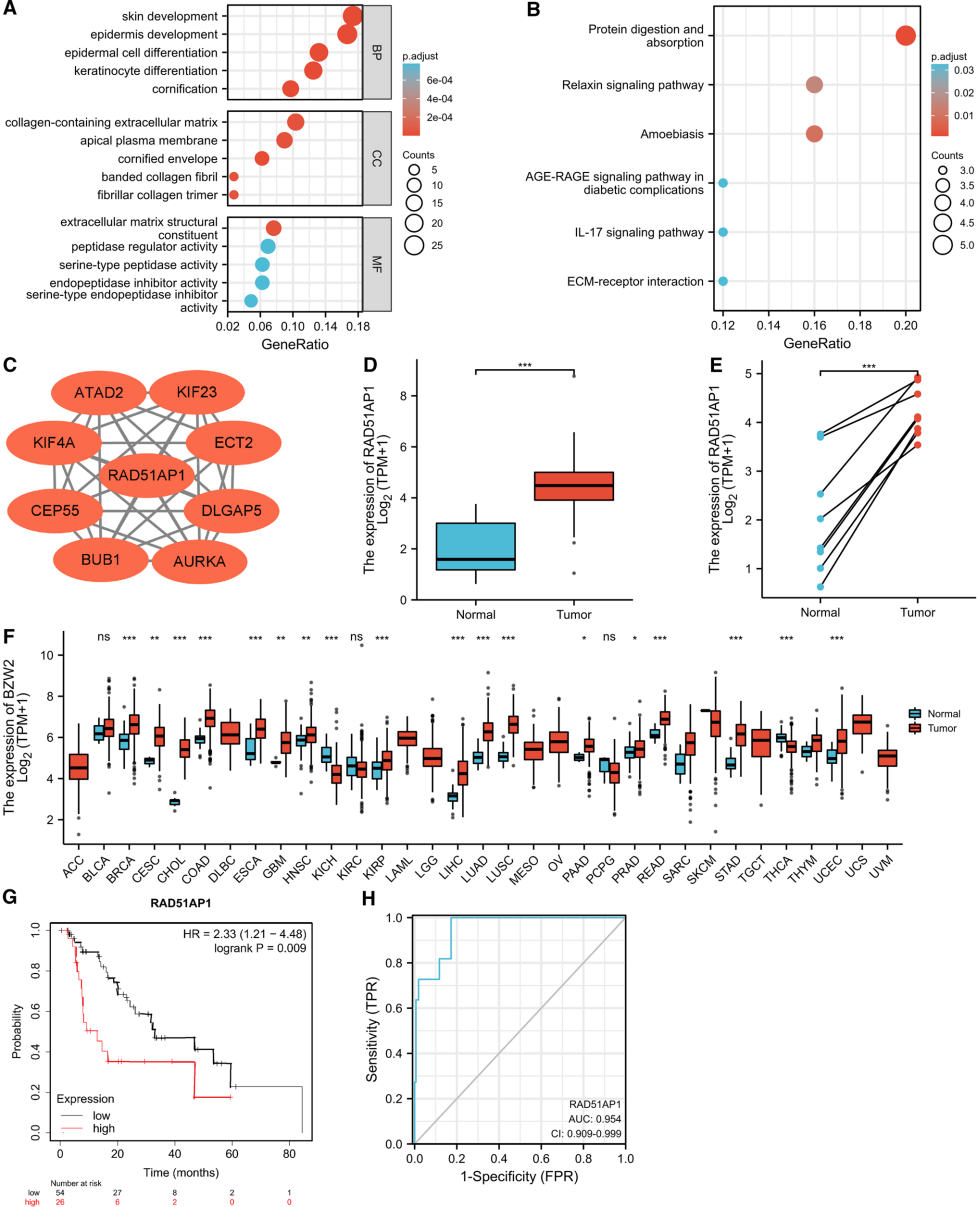
RAD51AP1 was identified as a targeted gene associated with poor prognosis in ESCC. **A** The GO functional analysis of overlapping DEGs. **B** The KEGG pathway analysis of overlapping DEGs. **C** The PPI network of hub genes obtained using the MCODE plugin with a total of 9 hub genes being identified in the submodule. **D** RAD51AP1 was overexpressed in ESCC tissues compared to normal tissues based on TCGA database. **E** RAD51AP1 was overexpressed in ESCC tissues compared to matched normal tissues. **F** The expression of RAD51AP1 in diverse cancer types. **G** High expression of RAD51AP1 was significantly associated with worse survival in ESCC patients. **H** The ROC curve of RAD51AP1 expression in ESCC. DEGs, differentially expressed genes; ESCC, esophageal squamous cell carcinoma; GO, Gene Ontology; KEGG, Kyoto Encyclopedia of Genes; PPI, protein–protein interaction; ROC, receiver operating characteristic. *p < 0.05; **p < 0.01, ***p < 0.001

GO functional analysis was used to explore potential biological roles of DEGs in ESCC, including Biological Process (BP), Cellular Component (CC), as well as Molecular Function (MF). For BP, DEGs were mainly enriched in skin development, epidermis development, epidermal cell differentiation, keratinocyte differentiation and cornification. For CC, DEGs were significantly enriched in collagen-containing extracellular matrix, apical plasma membrane, cornified envelope, banded collagen fibril and fibrillar collagen trimer. For MF, DEGs were obviously enriched in extracellular matrix structural constituent, peptidase regulator activity, serine-type peptidase activity, endopeptidase inhibitor activity and serine-type endopeptidase inhibitor activity (Fig. 2A). We then conducted KEGG enrichment analysis to find pivotal signaling pathways associated with DEGs. The results revealed that DEGs were significantly enriched in protein digestion and absorption, relaxin signaling pathway, amoebiasis, AGE-RAGE signaling pathway in diabetic complications, IL-17 signaling pathway, and ECM-receptor interaction (Fig. 2B).

### PPI network construction and hub gene identification

The PPI network of DEGs was constructed by STRING database and was visualized in Cytoscape software. One module of the PPI network was obtained using the MCODE plugin, the hub genes were CEP55, RAD51AP1, BUB1, KIF4A, DLGAP5, ECT2, ATAD2, KIF23, and AURKA (Fig. 2C). Existing literature indicated that RAD51AP1 has been poorly studied in ESCC. Therefore, RAD51AP1 was selected for our subsequent research. The transcriptional expression level of RAD51AP1 in pan-cancer was firstly analyzed by using RNA-seq data from TCGA database. The results revealed that RAD51AP1 was significantly upregulated in almost all tumor types, including ESCC (Fig. 2D–F). We then evaluated the prognostic value of RAD51AP1 in ESCC. As shown in Fig. 2G, high expression of RAD51AP1 was associated with worse survival in ESCC. RAD51AP1 expression also showed good diagnostic predictive ability, as the ROC curve exhibited that the expression of RAD51AP1 in ESCC was 0.954 (95% CI: 0.909–0.999) (Fig. 2H).

### The correlation between RAD51AP1 expression and immune infiltration

**Fig. 3:**
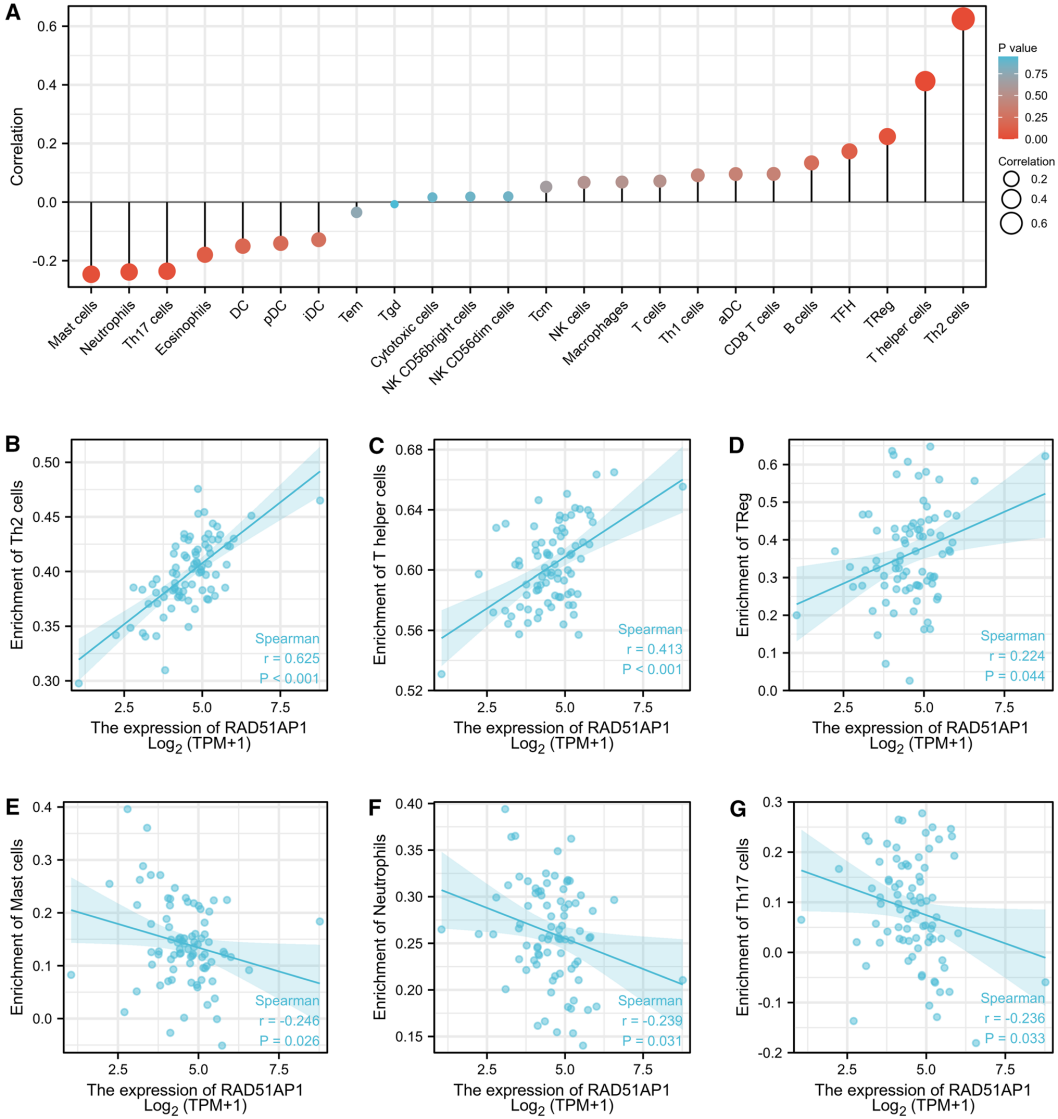
The correlation between RAD51AP1 expression and immune filtration level in ESCC. **A** The correlation between infiltration level of immune cells and RAD51AP1 expression in ESCC. **B**–**D** RAD51AP1 expression was positively associated with the enrichment of Th2 cells, T helper cells, and Treg cells. **E**–**G** RAD51AP1 expression was negatively associated with the enrichment of Mast cells, Neutrophils cells and Th17 cells

The relationship between the RAD51AP1 mRNA expression and the infiltration level of immune cells were further analyzed (Fig. 3A). The results showed that RAD51AP1 mRNA expression was positively associated with the enrichment of Th2 cells (r = 0.625, P < 0.001, Fig. 3B), T helper cells (r = 0.413, P < 0.001, Fig. 3C) and Treg cells (r = 0.224, P = 0.044, Fig. 3D). In addition, RAD51AP1 mRNA expression was negatively associated with the enrichment of Mast cells (r = − 0.246, P = 0.026, Fig. 3E), Neutrophils cells (r = − 0.239, P = 0.031, Fig. 3F) and Th17 cells (r = − 0.236, P = 0.033, Fig. 3G). In summary, these findings partially supported that RAD51AP1 participated in the immune response in ESCC tumor microenvironment.

### Knockdown of RAD51AP1 inhibited proliferation of ESCC cells

**Fig. 4:**
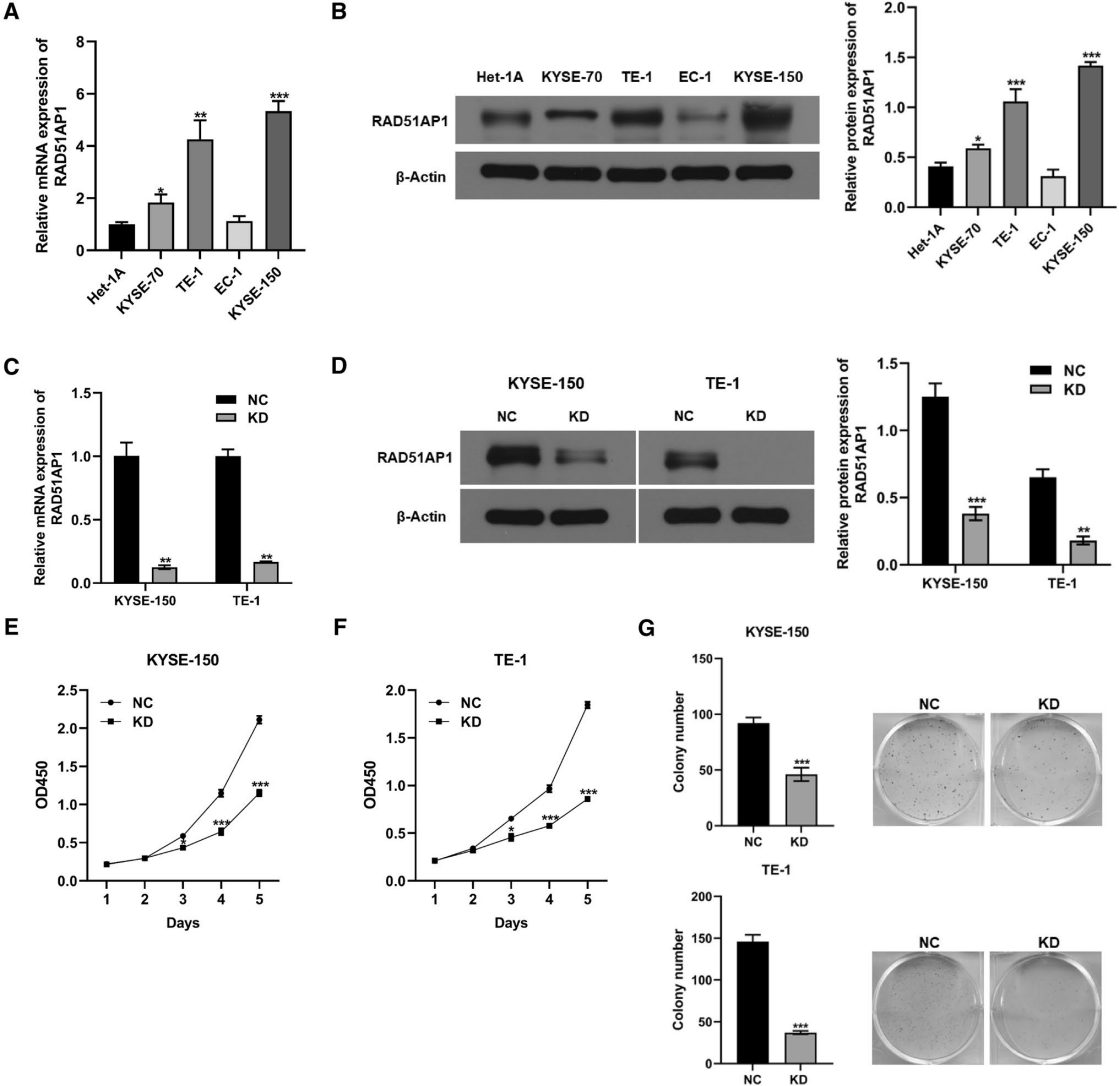
Knockdown of RAD51AP1 inhibited cell proliferation in ESCC. **A**, **B** QPCR and western blot analyses of RAD51AP1 mRNA and protein expression in ESCC cell lines (KYSE70, KYSE150, TE-1, and EC-1) and esophageal epithelial cell line (Het-1A). **C**, **D** QPCR and western blot analyses of KYSE150 and TE-1 cells transfected with RAD51AP1 shRNA and negative control. **E**, **F** CCK-8 assay showed knockdown of RAD51AP1 significantly inhibited cell proliferation in KYSE150 and TE1 cells. **G** Colony formation assay showed knockdown of RAD51AP1 significantly decreased colony number of KYSE150 and TE1 cells. ESCC, esophageal squamous cell carcinoma. *p < 0.05; **p < 0.01, ***p < 0.001

QPCR and western blot analyses were conducted to measure the mRNA and protein expression levels of RAD51AP1 in ESCC cell lines (KYSE70, KYSE150, TE-1, and EC-1) and esophageal epithelial cell line (Het-1A). As shown in Fig. 4A, B, the mRNA and protein expression of RAD51AP1 were significantly higher in KYSE70, KYSE150, and TE-1 cells compared to Het-1A cells. Therefore, KYSE150 and TE-1 cell lines were selected for subsequent experiments. To explore the potential role of RAD51AP1 in ESCC progression, RAD51AP1 shRNA (KD) or negative control (NC) was transfected into KYSE150 and TE1 cells. The knockdown efficiency was validated by qPCR and western blot assays (Fig. 4C, D).

We further investigated the effect of RAD51AP1 on cell proliferation, and CCK-8 assay revealed that knockdown of RAD51AP1 notably decreased the cell proliferative ability of KYSE150 and TE1 cells (Fig. 4E, F). Furthermore, results from colony formation assay showed that the colony number of KYSE150 and TE1 cells in KD group were significantly lower than that in NC group (Fig. 4G).

### Knockdown of RAD51AP1 induced cell cycle arrest and promoted cell apoptosis

**Fig. 5:**
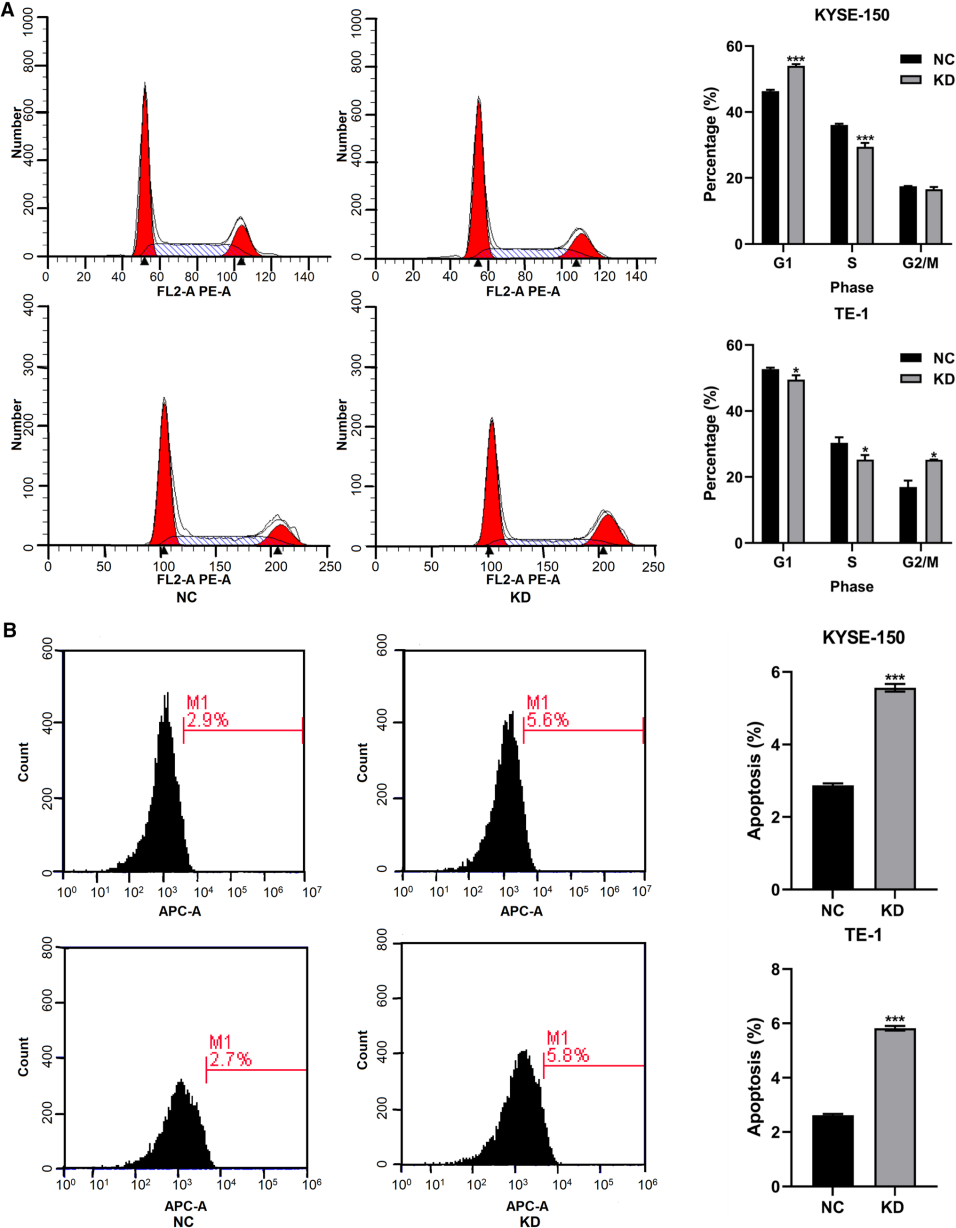
Knockdown of RAD51AP1 induced cell cycle arrest and cell apoptosis in ESCC. **A** Flow cytometry analysis showed that knockdown of RAD51AP1 led to cell cycle arrest in KYSE150 and TE1 cells. **B** Flow cytometry analysis showed that knockdown of RAD51AP1 markedly promoted cell apoptosis in KYSE150 and TE1 cells. ESCC, esophageal squamous cell carcinoma. *p < 0.05; **p < 0.01, ***p < 0.001

To determine whether knockdown of RAD51AP1 inhibited ESCC cell proliferation via regulating cell cycle and cell apoptosis, we examined cell cycle distribution and cell apoptosis level by using flow cytometry analysis. Our data indicated that knockdown of RAD51AP1 led to cell cycle arrest at the G0/G1 phase and G2/M phase in KYSE150 and TE1 cells, respectively (Fig. 5A). In addition, the percentage of apoptotic cells was significantly increased in RAD51AP1 knockdown group compared with negative control group (Fig. 5B).

### Knockdown of RAD51AP1 suppressed migration and invasion of ESCC cells

**Fig. 6:**
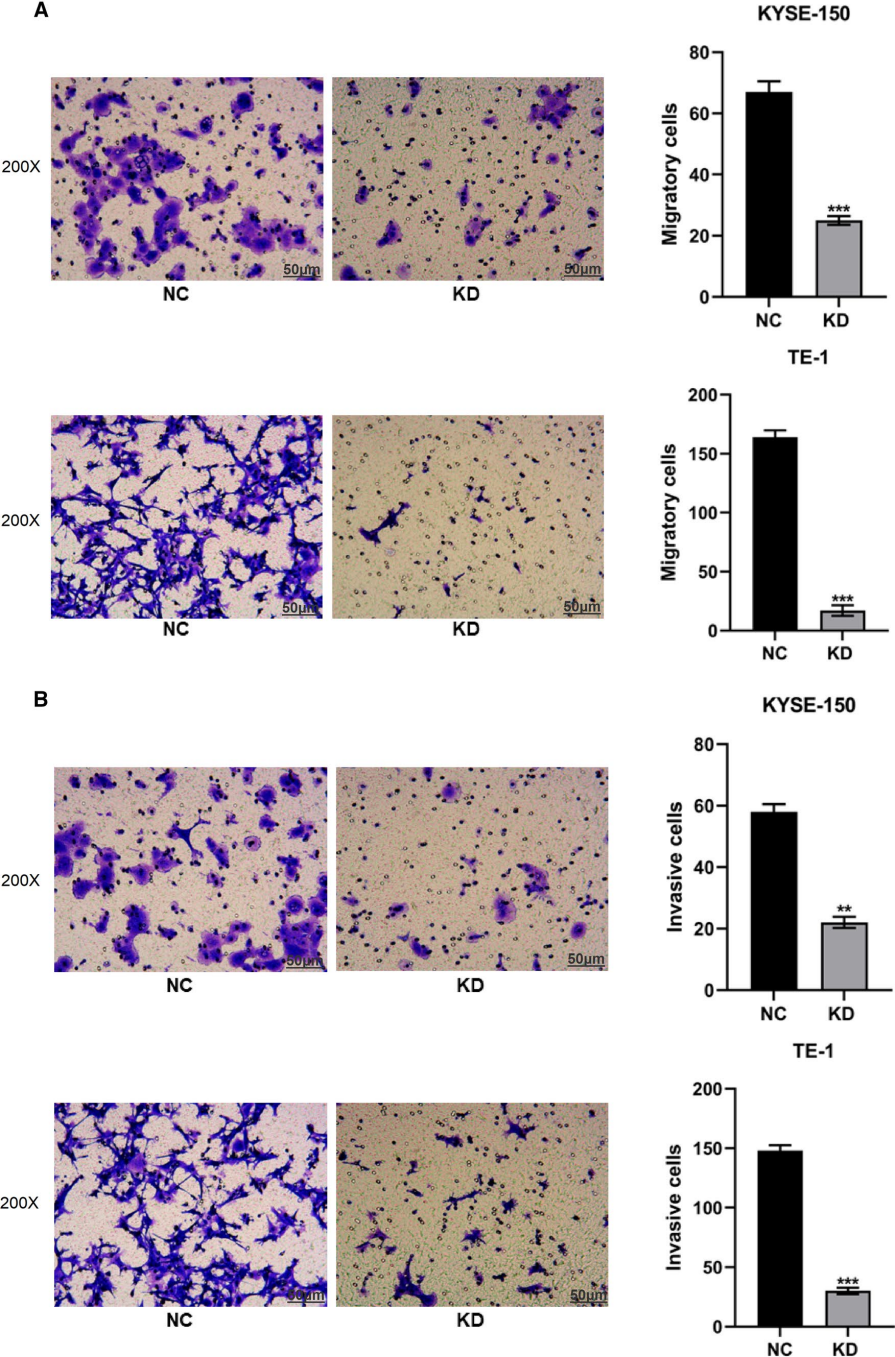
Knockdown of RAD51AP1 suppressed cell migration and invasion of in ESCC. **A** Transwell assay showed that knockdown of RAD51AP1 significantly inhibited cell migration in KYSE150 and TE1 cells. **B** Transwell assay showed that knockdown of RAD51AP1 significantly inhibited cell invasion in KYSE150 and TE1 cells. ESCC, esophageal squamous cell carcinoma. *p < 0.05; **p < 0.01, ***p < 0.001

Tumor metastasis is the most life-threatening aspect of ESCC. We then detected whether RAD51AP1 influenced the migratory and invasive abilities of ESCC cells through transwell assays. The results demonstrated that knockdown of RAD51AP1 significantly suppressed cell migration as well as cell invasion in KYSE150 and TE1 cells (Fig. 6A, B). Taken together, our data suggested that RAD51AP1 played an important role in regulating the proliferation and invasion of ESCC cells.

## Discussion

ESCC is the predominant type of esophageal cancer all over the world. A number of ESCC patients are diagnosed at an advanced clinical stage owing to the deficiency of effective biomarkers, and the prognosis of ESCC is still poor [18]. Therefore, looking for more efficient biomarkers for the diagnosis and prognosis of ESCC is urgently required. In the present study, bioinformatic analyses were performed by integrating three datasets from the GEO database. A total of 150 overlapping DEGs were identified, and 9 hub genes were screened from PPI network by using the MCODE plugin. The survival analysis showed that the expression of RAD51AP1 was significantly correlated with the prognosis of esophageal cancer. Furthermore, in a series of cellular functional experiments, knockdown of RAD51AP1 inhibited ESCC cell proliferation through inducing cell cycle arrest and cell apoptosis. Knockdown of RAD51AP1 was also found to decrease the migratory and invasive abilities of ESCC cells. Above of all, our results indicated that RAD51AP1 might act as an oncogene in ESCC.

Previous studies have reported that RAD51AP1 is dysregulated in various types of cancer. Chudasama et al. found that RAD51AP1 was significantly overexpressed in lung as well as ovarian cancer patients in both tissue and blood samples compared with healthy controls, and KM plots predicted worse OS in patients with high expression of RAD51AP1 [19]. Another study demonstrated that RAD51AP1 mRNA expression was significantly upregulated in tumor tissues compared to adjacent tissues in patients with hepatocellular carcinoma, and upregulation of RAD51AP1 expression was associated with advanced tumor stage, intrahepatic metastasis, vascular invasion and AFP level elevation [10]. In the present study, we consistently showed that RAD51AP1 expression was markedly increased in the ESCC tissues from data in the TCGA database and high expression of RAD51AP1 was related to poor prognosis of ESCC patients, suggesting that RAD51AP1 might be a potential biomarker for the prognosis of ESCC.

Numerous studies have confirmed that tumor immune cell infiltration was significantly correlated with tumor progression, and have a remarkable impact on immunotherapy, chemotherapy, and prognosis of patients with cancer [20–22]. RAD51AP1 was reported to be associated with immune infiltration of ovarian cancer, and upregulation of RAD51AP1 accompanied by accumulated Th2 cells, but decreased CD4 + T cells and CD8 + T cells [7]. To explore the potential role of RAD51AP1 in the tumor microenvironment of ESCC, we assessed the associations between the expression of RAD51AP1 and the infiltration level of immune cells. The results showed that RAD51AP1 expression was significantly positively correlated with the infiltration levels of Th2 cells, T helper cells and Treg cells. Th2 cells and Treg cells were considered as the oncogenic immune cells implicated in several types of tumors [23–26]. For example, the infiltration of Th2 cells promote tumor progression, and significantly correlates with poor survival in pancreatic cancer [27]. In addition, Lu et al. found that the proportion of Treg cells is increased in human gastric cancer and related to bad outcome [28]. In this study, Th2 cells and Treg cells were both elevated, which suggested that RAD51AP1 might contribute to mediate the immune escape of ESCC.

As mentioned above, high expression of RAD51AP1 might contribute to tumor progression of ESCC. A series of cellular functional experiments were then performed to study how RAD51AP1 involved in the development of ESCC. The results revealed that downregulation of RAD51AP1 could decrease cell proliferation, induce cell cycle arrest and enhance cell apoptosis in ESCC. Similarly, Bridges et al. reported that RAD51AP1 silencing in breast cancer cell lines dramatically reduced tumor growth [9]. Furthermore, inhibition of RAD51AP1 by short interfering RNA resulted in the cell growth suppression of cholangiocarcinoma [29]. In addition, our data demonstrated that knockdown of RAD51AP1 significantly suppressed cell migration and invasion in ESCC. Wu et al. expressed that RAD51AP1 silencing restrained the epithelial-mesenchymal transition and metastasis of non-small cell lung cancer [8]. Taken together, these findings indicated that RAD51AP1 could regulate cell growth and metastasis to promote the tumor development of ESCC in vitro.

## Conclusions

In summary, we firstly provided evidence that RAD51AP1 was overexpressed in ESCC, and upregulation of RAD51AP1was significantly associated with poor survival and immune infiltration. Additionally, our experiments demonstrated that knockdown of RAD51AP1 markedly suppressed cell proliferation and invasion in ESCC. Therefore, RAD51AP1 might be a potential therapeutic target for ESCC in the future.

## Data Availability

The datasets used and/or analyzed during the current study are available from the corresponding author on reasonable request.

## Abbreviations

ESCC: Esophageal squamous cell carcinoma
GEO: Gene Expression Omnibus
DEGs: Differentially expressed genes
GO: Gene Ontology
KEGG: Kyoto Encyclopedia of Genes
PPI: Protein–protein interaction
RAD51AP1: RAD51-associated protein 1
TCGA: The Cancer Genome Atlas
ssGSEA: Single-sample gene set enrichment analysis
shRNA: Short hairpin RNA
qPCR: Quantitative real-time PCR
CCK-8: Cell counting kit-8
PI: Propidium iodide
BP: Biological processes
CC: Cellular components
MF: Molecular functions
